# Himalayan Origin and Evolution of *Myricaria* (Tamaricaeae) in the Neogene

**DOI:** 10.1371/journal.pone.0097582

**Published:** 2014-06-06

**Authors:** Ming-Li Zhang, Hong-Hu Meng, Hong-Xiang Zhang, Byalt V. Vyacheslav, Stewart C. Sanderson

**Affiliations:** 1 Key Laboratory of Biogeography and Bioresource in Arid Land, Xinjiang Institute of Ecology and Geography, Chinese Academy of Sciences, Urumqi, China; 2 Institute of Botany, Chinese Academy of Sciences, Beijing, China; 3 Komarov Botanical Institute, Russian Academy of Sciences, St. Petersburg, Russia; 4 Shrub Sciences Laboratory, Intermountain Research Station, Forest Service, U.S. Department of Agriculture, Utah, United States of America; University of York, United Kingdom

## Abstract

**Background:**

*Myricaria* consists of about twelve-thirteen species and occurs in Eurasian North Temperate zone, most species in the Qinghai-Tibet Plateau (QTP) and adjacent areas.

**Methodology/Principal Findings:**

Twelve species of *Myricaria* plus two other genera *Tamarix* and *Reaumuria* in Tamaricaceae, were sampled, and four markers, ITS, *rps*16, *psb*B-*psb*H, and *trn*L-*trn*F were sequenced. The relaxed Bayesian molecular clock BEAST method was used to perform phylogenetic analysis and molecular dating, and Diva, S-Diva, and maximum likelihood Lagrange were used to estimate the ancestral area. The results indicated that *Myricaria* could be divided into four phylogenetic clades, which correspond to four sections within the genus, of them two are newly described in this paper. The crown age of *Myricaria* was dated to early Miocene ca. 20 Ma, at the probable early uplifting time of the Himalayas. The Himalayas were also shown as the center of origin for *Myricaria* from the optimization of ancestral distribution. Migration and dispersal of *Myricaria* were indicated to have taken place along the Asian Mountains, including the Himalayas, Kunlun, Altun, Hendukosh, Tianshan, Altai, and Caucasus etc., westward to Europe, eastward to Central China, and northward to the Mongolian Plateau.

**Conclusions/Significance:**

*Myricaria* spatiotemporal evolution presented here, especially the Himalayan origin at early Miocene ca. 20 Ma, and then migrated westward and eastward along the Asian mountains, offers a significant evolutionary case for QTP and Central Asian biogeography.

## Introduction

**Figure 1:**
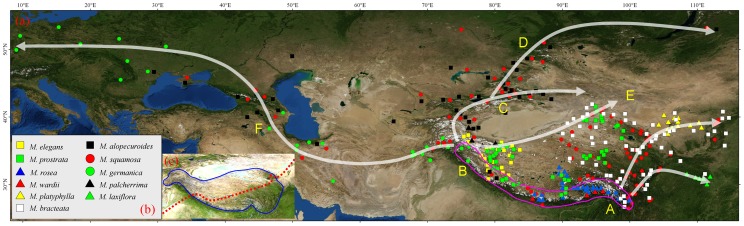
Distribution of *Myricaria* species (a,b), the information obtained from floras and herbaria, mainly in China. Geographical division of QTP eastern and western portions is shown in a red broken line (c). The Himalayan origin and dispersal routes along the Asian mountains are illustrated in arrows (a).

The Tamaricaceae contains about eighty species [1] and four genera: *Tamarix, Myricaria, Reumuria,* and *Hololachna* [2]. This family, and Frankeniaceae, are defined as the salt-gland anatomical lineage [3]. *Myricaria* consists of about twelve - thirteen species [4]–[7] and occurs in Northern Temperate zone of Eurasia, mainly along the Asian mountains. There are eight species in Himalayas, many are endemic, thus forming a center of diversity for *Myricaria* (see Figure 1).

Desvaux (1825) established the genus *Myricaria* and Niedenzu [8] presented the first classification. Zhang & Zhang [4] studied *Myricaria* in China and recognized ten species; they presumed the Himalayas to be the center of origin based mainly on the distribution of species. Gorschkova [7] described six species belonging to two sections in the flora of the former USSR.

Another issue relevant to *Myricaria* systematics is the species *Myricaria elegans*. Ovezinrlikov & Kinzikaeva [9] erected the genus *Myrtama* based on this species but it caused some controversy. Zhang *et al.* [10] used ITS sequence data to study the relationships within Tamaricaceae and regarded *Myrtama* as an intermediate genus between *Myricaria* and *Tamarix* [11]. After sampling four species from *Myricaria* and sequencing ITS, *rbc*L, and tRNAs Ser (GCU) and Gly (UCC), Gaskin *et al.* [1] found *Myrtama* and *Hololachna* to be distinct within Tamaricaceae, as did Zhang *et al.* [10]. However, based on additional sequence data, Hua *et al.* [12] and Wang *et al*. [13] confirmed that *Myrtama* should be included in *Myricaria*. Sampling ten species of *Myricaria* and sequencing cpDNA *psb*A–*trn*H and the *rpL*16 intron, Liu *et al.* [14]–[15] investigated the species-level phylogeographical patterns of *Myricaria* in western China as well as the origin of *M. laxiflora*, a unique subtropical species of conservation concern from the Three Gorges of the Yangtze River in Sichuan and Hubei provinces. The Himalayas were proposed as the center of origin of *Myricaria* by Liu *et al*. [14] with the estimated age of origin 1.46–2.30 Ma.

Closely associated with the distribution pattern of *Myricaria* and related taxa, the QTP and Himalayan uplift during the Neogene are hypothesized to be a major influence on organism evolution in Asia (e.g. [16]–[17]). Following collision of the Indian and Eurasian continents at ca. 50 Ma, the altitude and range of the QTP near the Oligocene-Miocene boundary became sufficient to trigger a reorganization of the Asian climate, as evidenced by the beginning of loess deposition in the Chinese Loess Plateau and the Junggar Basin [18]–[20]. Some evidence confirms that the central areas of the QTP were raised to present altitudes by that time [16], [21]–[22] and uplift of the Himalayas may have also begun at that time [22]–[23]. Uplift of peripheral portions of the plateau has continued at various intervals [24]–[28]. A major uplift of QTP is often suggested to have occurred at 8 Ma, which also coupled with global cooling, even though Molnar [29] considered this uplift evidence to be inconclusive. Uplift of the QTP and global climate cooling and aridification [27] have been suggested causes for the evolution of many organisms [30]–[36]. As these studies have shown, rapid diversification of lineages in the QTP resulted in the migration of some species into other temperate regions, such as Central Asia, the Arctic, the Mediterranean (Caucasus-Alps) and southern Asia. Of these, connections between the QTP and adjacent arid, more northern areas can often be discerned, for example in recent studies on *Hippophae rhamnoides* (Elaeagnaceae) [37], *Caragana* (Fabaceae) [38], and *Astragalus* (Fabaceae) [39]. Linkage of the QTP and Africa and/or the Mediterranean is illustrated by *Begonia* [40] and *Uvaria* (Annonaceae) [41]. An example linking the QTP and Southeast Asia is *Paini* (Anura: Dicroglossidae) [42].

The origin of *Myricaria* has been associated with the QTP and Himalayas but justification has been weak. Zhang & Zhang [4] presumed that *Myricaria* originated from the Himalayas, only based on species distribution of the genus, whereas same opinion conducted by Liu *et al.* [14] from a phylogeography. Here we attempt to examine the origin and evolution of this genus and link it to the Himalayan uplift to explain the causes of its evolutionary patterns. In addition, the classification and distribution of *Myricaria* are examined using molecular phylogeny and biogeography.

## Materials and Methods

### Taxa sampled

Twelve species (seventeen samples) of *Myricaria* plus seven species from the outgroups *Tamarix* and *Reummuria* served as sources of DNA material (Table 1). The herbaria utilized in China were as follows: HNWP (Northwest Institute of Plateau Biology, Chinese Academy of Sciences (CAS), Xining, Qinghai); SHI (Shihezhi University, Shihezhi, Xinjiang); and XJBI (Xinjiang Institute of Ecology and Geography, CAS, Urumqi, Xinjiang), as well as the LE (Komarov Botanical Institute, Russian Academy of Sciences, St. Petersburg, Russia).

**Table 1:** List of Sampled Taxa, Vouchers and Genebank Accession Numbers.

Species	Voucher	Source	ITS	*trn*L-*trn*F	*rps*16	*psb*B-*psb*H
***Myricaria*** ** Desv.**						
*M. alopecuroides* Schrenk.	P. Yan 3650 (SHI)	Tashikurgan, Xinjiang, China, alt. 3650m	KJ729654	KJ729806	KJ729756	KJ729705
*M. alopecuroides* Schrenk. 1	Tibet-Xinjiang Exp. Team 1034 (HNWP)	Sukepiya, Yecheng, Xinjiang, China, alt. 2800m	KJ808603	KJ808634	KJ808619	KJ808649
*M. bracteata* Royle	Y.H. Wu 36461 (HNWP)	Nuomuhong, Dulan, Qinghai, China, alt. 2840m	KJ729655	KJ729807	KJ729757	KJ729706
*M. elegans* Royle 1	P. Yan 3999 (SHI)	Bandir, Tashekurgan, Xinjiang, China, alt. 3000m	KJ808604	KJ808635	KJ808620	KJ808650
*M. elegans* Royle 2	P. Yan 7178 (SHI)	Mazhaxi, Yecheng, Xinjiang, China, alt. 3600m	KJ808605	KJ808636	KJ808621	KJ808651
*M. elegans* Royle 3	P. Yan 7378 (SHI)	Ritu, Tibet, China, alt. 4600m	KJ808606	KJ808637	KJ808622	KJ808652
*M. germenica* (L.) Desv.	I.O. Baitulin, Aralbaiev s.n. (LE)	Zajsanskaya depression, E. Kazakistan	KJ808607	KJ808638	----	----
*M. laxiflora* (Franch.) P.Y. Zhang et Y.J. Zhang 1	Wuhan Bot Gard	Wuhan Bot Gard, Hubei, China	KJ808608	KJ808639	KJ808623	KJ808653
*M. laxiflora* (Franch.) P.Y. Zhang et Y.J. Zhang 2	Wuhan Bot Gard	Wuhan Bot Gard, Hubei, China	KJ808609	KJ808640	KJ808624	KJ808654
*M. paniculata* P.Y. Zhang et Y.J. Zhang	B.Z. Guo; W.Y. Wang 21930 (HNWP)	Linzhi, Tibet, China, alt. 2000m	KJ808610	----	----	KJ808655
*M. platyphylla* Maxim.	Z.Y. Yang; L.M. Ke 5711 (XJBI)	Houxia, Urumqi, Xinjiang, China	KJ808611	KJ808641	KJ808625	KJ808656
*M. prostrata* Hook.f. et Thomson ex Benth. et Hook.f.	P. Yan 7242 (SHI)	Hechakou, Hetian, Xinjiang, China, alt. 5000m	KJ808612	KJ808642	KJ808626	KJ808657
*M. pulcherrima* Batalin	L.M. Ke 121 (XJBI)	Ermuchang, Shaya, Xinjiang, China alt. 4350m	KJ808613	KJ808643	KJ808627	KJ808658
*M. rosea* W.W. Sm.	R.F. Huang G89-485 (HNWP)	Milinpaiqu, Tibet, China, alt. 4530m	KJ808614	----	KJ808628	KJ808659
*M. squamosa* Desv.	P. Yan 4002 (SHI)	Bandir, Tashekurgan, Xinjiang, China, alt. 3002m	KJ729658	KJ729810	KJ729760	KJ729709
*M. squamosa* Desv. 1	Y.H. Wu 3077 (HNWP)	Beishan, Huzhu, Qinghai, China, alt. 2700m	KJ808615	KJ808644	KJ808629	KJ808660
*M. wardii* C.Marquand Sun YX	R.H. Ree, S.K. Wu 30159 (LE)	Linzhi-Bomi, Tibet, China, alt. 3550m	KJ808616	KJ808645	KJ808630	KJ808661
***Reaumuria*** ** Linn.**						
*R. kaschgarica* Rupr. 1	Tibet-Xinjiang Exp. Team 5166 (HNWP)	Tashekurgan, Xinjiang, China	KJ808617	KJ808646	KJ808631	KJ808662
*R. kaschgarica* Rupr. 2	Y.M. Duan 84-A-012 (XJBI)	Ruoqiang, Xinjiang, China, alt. 3080m	----	KJ808647	KJ808632	KJ808663
*R. soongarica* (Pall.) Maxim.	Tibet-Xinjiang Exp. Team 5098(SHI)	Tashekurgan, Xinjiang, China, alt. 2300m	KJ808618	KJ808648	KJ808633	KJ808664
***Tamarix*** ** L.**						
*T. karakalensis* Freyn	K.B. Blinkovsky 12 VIII 1953 (LE)	C. Kopetdag, Ashkhabad, Turcominia	KJ729659	KJ729811	KJ729761	KJ729710
*T. laxa* Willd.	O.N. Demina 18 V 2001 (LE)	Orlovsky, Bostov, Russia	KJ729660	----	KJ729762	KJ729711
*T. meyeri* Boiss.	M.R. Tanybaeva 12 V 2007 (LE)	Turkestan Ridge, Kirgiztan	KJ729661	KJ729812	KJ729763	KJ729712
*T. ramosissima* Ledeb.	N.A. Brykova s.n. 10 VII 1998 (LE)	Orlovsky, Bostov, Russia	KJ729662	KJ729813	KJ729764	KJ729713

Herbaria: HNWP (Northwest Institute of Plateau Biology, Chinese Academy of Sciences, Xining, Qinghai, China); SHI (Shihezi University, Shihezi, Xinjiang, China), XJBI (Xinjiang Institute of Ecology and Geography, Chinese Academy of Sciences, Urumqi, Xinjiang, China); LE (Komarov Botanical Institute, Russian Academy of Sceinces, Russia, St. Petersburg, Russia).

### DNA sequencing

Total genomic DNA was extracted using the CTAB method [43]. The polymerase chain reaction (PCR) was used for amplification of double stranded DNA. The 25 µl reaction system contained 0.25 µl of Ex *Taq*, 2.5 µl of 10× Ex Taq buffer (Mg^2+^ concentration of 25 mM), 2.0 µl of dNTP mix (2.5 mM concentration for each dNTP), 1 µl of the forward and reverse primers at 5 umol/µl, and 0.5 µl of template DNA. The following primers were used: for ITS: ITS1-*f* (5′-AGA AGT CGT AAC AAG GTT TCC GTA GC-3′) and ITS4-*r* (5′-TCC TCC GCT TAT TGA TAT GC-3′), for *trn*L-*trn*F: *trn*L-*f* (5′-CGA AAT CGG TAG ACG CTA CG-3′) and *trn*F-*r* (5′-ATT TGA ACT GGT GAC ACG AG -3′), for the intron of *rps*16: *rps*16-*f* (5′-GTG GTA GAA AGC AAC GTG CGA CTT-3′), and for *rps*16-*r* (5′-TCG GGA TCG AAC ATC AAT TGC AAC-3′) [44]; and the intergenic spacer *psb*B-*psb*H: *psb*B-*psb*H-*r* (5′-TTCAACAGTTTGTGTAGCCA-3′) and *psb*B-*psb*H-*f* (5′-AGATGTTTTTGCTGGTATTGA-3′) [45].

The protocol for amplification consisted of an initial hotstart at 95°C for 2 min, followed by 30 cycles of denaturation at 94°C for 30 s, annealing at 52°C for 30 s, extension at 72°C for 90 s, and a final extension at 72°C for 10 min. PCR products were purified using the PEG precipitation procedure [46]. These were sequenced using an ABI Prism 3770 Genetic Analyzer (Shanghai Sanggon Biological Engineering Technology & Service, Shanghai, China). Sequences were aligned using CLUSTAL X software [47], and then adjusted by hand. All gaps were treated as missing characters. Finally, a combined dataset consisting of four sequences of ITS and three cpDNA *trn*L-*trn*F, *rps*16, and *psb*B-*psb*H, was prepared for phylogenetic analysis.

### Phylogenetic analysis and divergence time estimate

The sequence dataset from twelve species (seventeen samples) of *Myricaria* plus seven species of *Tamarix* and *Reummuria* yielded 3202 aligned nucleotide characters from four genes: ITS, *trn*L-*trn*F, *rps*16, and *psb*B-*psb*H. The incongruence length difference (ILD) test of the four gene datasets was carried out using PAUP* [48], to assess potential conflicts between the different DNA fragments. This test was implemented with 100 partition-homogeneity test replicates, using a heuristic search option with simple addition of taxa, TBR branch swapping and MaxTrees set to 1000. 0.222 of incongruence length difference (ILD) tests [48] showed that the four gene datasets were not incongruent.

Estimation of phylogenetic relationships and divergence time was conducted using a Bayesian method implemented in BEAST 1.5.4, employing a relaxed clock model [49]–[50]. We used the uncorrelated lognormal molecular clock model with a Yule process for the speciation model, GTR+I+G for the substitution model (estimated for the dataset), and a normal distribution with SD of 1 as priors on the calibration nodes to accommodate for calibration uncertainty. Minimum ages for the two normal priors were constrained to the root of all taxa and a node respectively, the family Tamaricaceae 70 Ma, and genus *Tamarix* 25 Ma, with a detailed description as follows. A Markov chain Monte Carlo analysis was run for 50 million generations and sampled every 1,000 generations, and two independent runs were performed to confirm the convergence of the analysis. The stationarity of each run was examined using the effective sampling size of each parameter (>200). The last 40 million generations were used to construct the maximum clade credibility tree and the associated 95% highest posterior density distributions around the estimated node ages.

### Optimization of ancestral distributions

#### Tamaricaceae root constrained

Tamaricaceae is included in the order Caryophyllales [2], [51] and has no reliable macrofossil record. According to molecular dating [52]–[54], the divergence time of the order has been estimated as ca. 100 Ma. Tiffney [55] considered that the extant woody families originated during the Cretaceous to early Eocene, while herbaceous families appeared during the late Oligocene to Miocene. For instance, the woody families Ulmaceae and Fabaceae appeared at about 70 Ma [54], [56] and 70–60 (-50) Ma [54], [56], [57] respectively. Families related to the Tamaricaceae, such as Polygonaceae and Amaranthaceae/Chenopodiaceae, have an approximate age of ca. 65 Ma [58]. The two subfamilies of the Caryophyllaceae have an approximate age of 40–55 Ma according to the age of the inflorescence fossil *Caryophylloflora paleogenica*, and the family has an possible age of ca. 73 (60–80) Ma [59]–[60]. Even though the ancestor of the related families Tamaricaceae and Frankeniaceae has been dated to 43–30 Ma [56], Tamaricaceae itself has had variable dating results. Bell et al. dated it to (72-) 60–58 (-44) [54], while Wikstrom et al. placed it at 52–37 Ma [56], and Schuster et al. at (125-) 118.7–110 (-90.7) [61]. In the light of these estimates, a balanced age for Tamaricaeae could be suggested as about 70 Ma; this estimate was chosen as the family root for molecular dating.

The earliest reliable fossil record of *Tamarix* is Miocene, from the Yunnan province of China [62]. Most of the available fossils are from the Miocene, therefore, the genus might be hypothesized to have had an origin at least in early Miocene. However, considering its wide distribution in Europe, Africa, Asia, and North America, and our limited samples mainly from China, we conservatively assigned an age of late Oligocene-early Miocene, at ca. 25 Ma for *Tamarix*.

### Areas

In accordance with the distribution of *Myricaria* species along the Asian mountains (Figure 1), we divided the distribution into six areas, namely, A: the eastern Himalayas, including the eastern QTP, the Hengduan mountains, and northern and central China; B: the western Himalayas, including the western QTP and the Pamir-Alai, Kunlun-Altun, and Hendukosh mountains; C: the Tianshan mountains and Junggar-Turan deserts; D: Altai-Siberia; E: the Mongolian Plateau; and F: Asia Minor-Caucasus-Europe. These six areas are distinct in biodiversity, vegetation, and floristics [16]–[18], [30], [32], [36]–[38].

### Optimization of ancestral distributions

To infer biogeographical events, three methods were used: a parsimony-based procedure Diva [63], S-Diva [64] and a maximum likelihood-based DEC model (Lagrange; [65]–[66]). These three approaches are simultaneously considered so that to assess the relevant biogeographical processes, such as vicariance, dispersal, and extinction.

#### Diva

**Figure 2:**
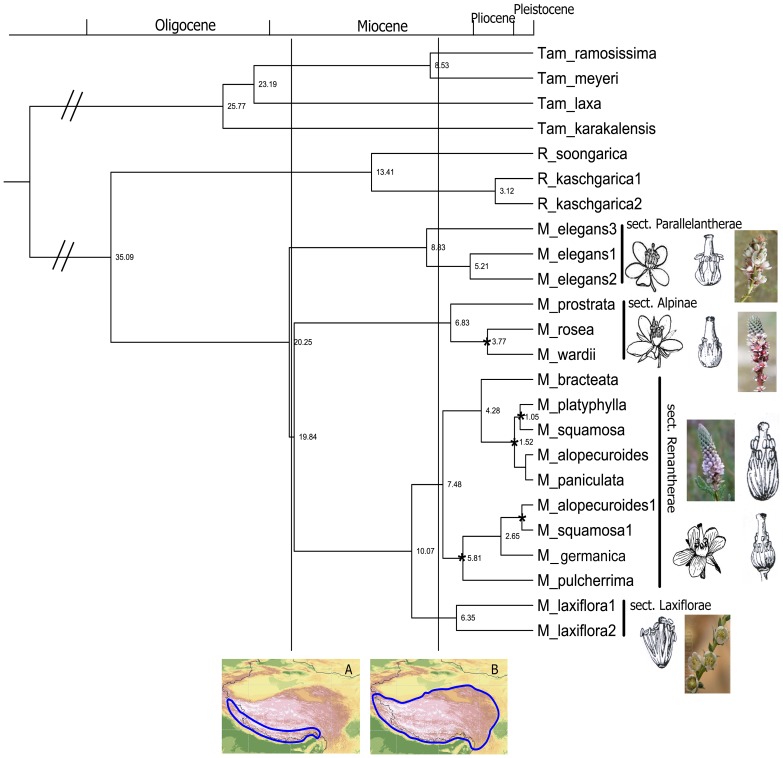
Chronogram of *Myricaria* and outgroups *Tamarix* and *Reaumuria* in Tamariaceae, with maximum clade credibility performed by BEAST. Dating values are plotted at the right of the nodes, and posterior probability support of more than 95% is labeled as “*” at the nodes. Two vertical lines are labeled at 20 Ma and 8 Ma, corresponding respectively to two stages and two high-altitude ranges (blue ranges in A, B) of the QTP uplift, Himalayan motion, and rapid and major-range uplift. Four sections within *Myricaria* are shown, along with flowers and degree of union of the filaments. In section *Alpinae*, flower and filament status refers to *M. rosea*, and in section *Renantherae*, the flower and filaments, above right, refer to *M. bracteata.* The filaments below left refer to *M. germanica* and those below right refer to *M. squamosa.*

Dispersal–vicariance analysis optimizes distributions for each node of the tree by minimizing the number of assumed dispersals and extinctions, and favors vicariance events [63], [67]. The Diva program reconstructs widespread ancestral distributions, restricting them to single areas. Because allopatric speciation by vicariance is the null model in Diva, vicariance and range division would always be the preferred explanation if the ancestors were widespread. To avoid inferring a widespread ancestor at the root because of the presence of widespread extant taxa, a limit of two areas was set (maxareas  =  2) in Diva [63]. The phylogenetic typology of the BEAST tree (Figure 2) was input for Diva analysis.

#### S-Diva

(or Bayes-Diva) [64] is a program which complements Diva and implements the methods of Nylander *et al*. [68] and Harris *et al.* [21], determining statistical support for ancestral range reconstructions using multiple trees from Bayesian analysis. This has the advantage that uncertainties in phylogenetic inference can be taken into account. One hundred Bayesian MCMC trees with the last stable typologies from BEAST, and a BEAST tree typology (Figure 2) were input into the S-Diva program.

#### Lagrange

A valuable new biogeographical method is parametric likelihood analysis, with a dispersal–extinction–cladogenesis model [67], as implemented in Lagrange v. 2.0.1 [65]. This method calculates the likelihood of biogeographical routes and areas occupied by the most recent common ancestor (MRCA) for a given phylogenetic tree topology (BEAST tree, Figure 2) and the present distribution of taxa. Therefore, dispersal and vicariance of lineages, represented by connection areas, can be estimated by the probabilities. This is thus a form of MRCA area reconstruction differing from the parsimony approach of Diva.

## Results

### Phylogenetic analysis and divergence time estimate

The phylogenetic tree obtained from Bayesian inference in BEAST showed that *Myricaria* is monophyletic and *Myrtama* should be included in *Myricaria* rather than treated as a distinct genus (Figure 2). Within *Myricaria*, four clades were recognized, two corresponding to the existing sections *Parallelantherae* Ndz. and *Renantherae* Ndz, the other two represent new groups to be named as sections *Alpinae* and *Laxiflorae* (see Appendix S1). Flowers and filaments of the plants are illustrated in Figure 2, to show the characteristics of the four sections. In the present phylogenetic tree, the clades of the genus and the sections have strong support, confirming the validity of taxa at the ranks of genus and section. Section *Renantherae* comprises the most species in the genus, and has two subclades. The two widely distributed species of this section, *M. alopecuroides* and *M. squamosa,* are located in each subclade. The crown age of *Myricaria* was ca. 20 Ma, and 8.83∼6.35 Ma for four sections.

### Optimization of ancestral distributions

**Figure 3:**
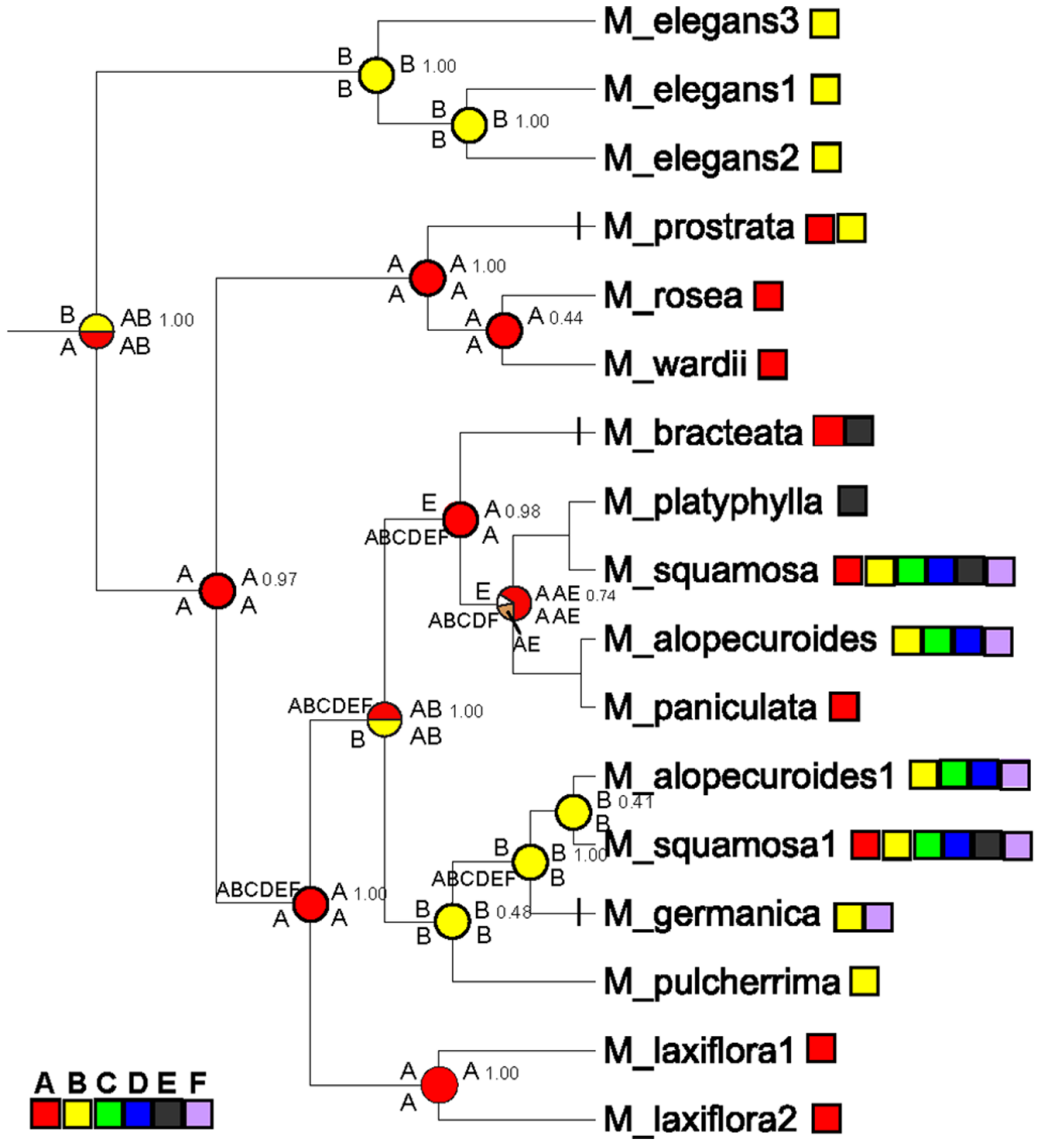
Biogeographical ancestral optimization performed with Diva, S-Diva and Lagrange. Pie charts at the internal nodes represent the calculated probabilities (relative frequencies) of alternative ancestral areas (reconstructions), and were produced by S-Diva; letters are labeled at the right and above nodes, and probabilities from S-Diva are at the right and below nodes; letters above and below at branches are from Lagrange, indicating the highest probability migration routes and inheritance of area by upper and lower descendant branches, respectively. Located at the front of *M. prostrata*, *M. bracteata*, and *M. germanica*, vertical lines on the branches indicate dispersals involving these three species. Area letters as stated in the text: **A**: eastern Himalayas, including the eastern QTP, Hengduan mountains, northern China, and central China of East Asia; **B**: western Himalayas, including the western QTP, and Pamir-Alai, Kunlun-Altun, and Hendukosh mountains; **C**: Tianshan-Tungger-Turan; **D**: Altai-Siberia; **E**: Mongolian Plateau; and **F**: Asia Minor-Caucasus-Europe.

The results of the three approaches Diva, S-Diva, and Lagrange (Figure 3) showed a consistent and strongly supported pattern, particularly at the ancestral nodes for *Myricaria* (AB), and among the four sections, *Parallelantherae* (B), *Alpinae* (A), *Laxiflorae* (A), and *Renantherae* with AB from Diva and S-Diva, whereas only with ABCDEF/B from Lagrange. On the whole, AB, A, and B, namely the Himalayas and the QTP, should be considered as ancestral areas in *Myricaria*. The events occurring in areas C, D, E, and F, were considered to be dispersals, several of which can be distinguished. *M. prostrata* occurs in the Himalayas, and its western Himalayan distribution was indicated to be a dispersal event from the eastern Himalayas. *M. bracteata* in sand lands of the Mongolian Plateau was shown to be a migrant from the eastern Himalayas, Hengduan Mountains, and Northern China. Whereas the distribution of *M. germanica* in Asia Minor-Caucasus-Europe was came from the western Himalayas.

## Discussion

### Phylogenetic division of sections within *Myricaria*

Niedenzu [8] divided *Myricaria* taxa into two sections: *Parallelantherae* Ndz. and *Renantherae* Ndz. Gorschakova [7] accepted this classification system in the Flora of the USSR. Zhang & Zhang [4], however, considered that the establishment of infrageneric ranks was not appropriate due to its complicated and variable morphological characters. Therefore, in the Flora of China [5]–[6] there is no division of infrageneric sections.

However, our phylogenetic tree (Figure 2) yielded a clear phylogenetic division of four sections, including two that are new. Of them the Himalayan and QTP section *Alpinae*, containing *M. prostrata, M. rosea,* and *M. wardii,* is characterized by the prostrate and recumbent, and with an adaptation to high altitudes of 3000–5300 m. Section *Laxiflorae*, comprises of only species *M. laxiflora*, endemic to the subtropical area of the Sichuan and Hubei provinces in eastern China. Detailed descriptions of two new sections are given in Appendix S1.

*Myricaria elegans* Royle was originally described from the Kunawar region of the western Himalayas [69]. Based on this species and its characters of 10 stamens, flat leaves, and no obvious style, the genus *Myrtama* was established [9]. Qaiser & Ali [70] named it *Tamaricaria*, whereas Baum [71] moved *Myricaria elegans* to *Tamarix* as *T. ladachensis*. As mentioned, Zhang *et al*. [10] and Gaskin *et al*. [1] accepted *Myrtama* at generic rank. Zhang *et al*. [10] considered that *Myricaria elegans* was an intermediate and hybrid genus between *Myricaria* and *Tamarix*, related more to *Tamarix*. However, Hua *et al*. [12] and Wang *et al*. [13], based on sequence data, found that it would be appropriately placed in *Myricaria*. Our results (Figure 1) also show that inclusion of *Myricaria elegans* in *Myricaria* is suitable, since the whole of *Myricaria*, including *Myricaria elegans,* has strong support (100%) (Figure 2). This is in accordance with evidence from morphological classification [4] and molecular phylogeny [12]–[14]. The former conclusion supporting retention of *Myrtama* [10] was only based on ITS sequence data, which is probably not sufficient evidence [72]. While in the phylogeny of Gaskin *et al*. [1] (their Figure 2), only four species were sampled, and four species as clade had a strong bootstrap support (99%) for the inclusion in *Myricaria* [1].

### Himalayan origin, ancestral inheritance, and multi-diversification in the Himalayas

Our estimated crown age of ca. 20 Ma for *Myricaria* (Figure 2) falls into the probable early range of the Himalayan uplift in early Miocene [22], consequently allows us to speculate that uplift of this mountain range caused the origin of *Myricaria*. The biogeographical analytical result of Diva, S-Diva and Lagrange, showing the combined Himalayan area AB as the ancestral area for *Myricaria* (see Figure 3), which also supports a Himalayan origin. The present molecular dating results are in contrast to previous phylogeographical opinion of a main divergence event at the implausible age of 1.46–2.30 Ma in the Plio-Pleistocene [14].

The southern and northern slopes of the Himalayas differ dramatically in temperature and precipitation. *Myricaria* species occurring on the northern slope are generally xeric, same as those of the main plateau [73]. The western portions of the Himalayas and adjacent QTP are more arid than the eastern parts [27], [73], see Figure 1c. Probably these differences are the cause of the persistent diversification between the eastern (A) and western Himalayas (B) for the *Myricaria* lineages (Figures 1 and 3). The eastern A contains the alpine species *M. rosea,* and *M. wardii*, and the western B includes *M. elegans* and *M. prostrata*. These four species are endemic to the Himalayas, and occupy two of the four *Myricaria* sections. In particular, the western Himalayas (B) is an important geographical node and dispersal center for *Myricaria,* with movement toward the Pamir-Alai, Hendukosh, Tianshan, and Kunlun-Altun mountains, etc. Noticeable, the Himalayas as a union was divided into A and B two times (see Figure 3), once at the time of generic origin and diversification, and the other at the diversification node of the sections *Renantherae* and *Laxiflorae*. Overall, the Himalayan areas AB, A, and B as the ancestors occurred at least seven times at the nodes in Figure 3. In detail, the Himalayan union AB as an ancestral area appeared at two nodes, with estimated ages of 20.25 Ma (genus crown age) and 10.07 Ma, the eastern Himalayas A at three nodes with ages of 19.84 Ma, 6.83 Ma (section *Alpinae* crown age), and 6.35 Ma (section *Laxiflorae* crown age), and the western Himalayas B occupied two, with ages of 8.83 Ma (section *Parallelantherae* crown age) and 5.81 Ma. All of these stressed the diversification and geographical heritage of Himalayan ancestry.

Many plant groups of the Northern Temperate Zone are hypothesized to have originated from the QTP [16], [30]–[36], especially from the Hengduan Mountains; few have originated from the Himalayas. *Myricaria* presents a case of Himalayan montane origin.

In some cases, the Himalayas are regarded as a corridor of plant species migration linking East Asia and Central Asia and/or the Mediterranean [16], [35], [74]–[77]. The Himalayas have acted as a migration corridor for Sino-Japanese elements westward and Mediterranean elements eastward, such as *Hippophae rhamnoides* (Elaeagnaceae) [37], *Pogonophace* (*Astragalus*, Fabaceae) [72], and *Phyllolobium* [77] (Fabaceae). They also served as a migration route westwards to Central Asia from East Asia for *Caragana* (Fabaceae) [78], *Begonia* migration was through the Himalayas eastwards from Africa [40]. Our *Myricaria* analysis shows endemism, origin, and remarkable multiple diversifications within the Himalayas, but with weak or absent migration here. The Himalayas therefore seems to serve as the center of origin for *Myricaria* rather than as a corridor. This is rather like as these QTP endemic plant groups, such as *Nannoglottis* (Asteraceae) [79], *Ligularia* complex (Asteraceae) [80], *Saussurea* (Asteraceae) [81], *Dolomiaea, Diplazoptilon* and *Xanthopappus* (Asteraceae) [82], and *Aconitum* (Ranunculaceae) [83], all of them are hypothesized to have originated from native QTP during Miocene-Pliocene.

### Diversification of *Myricaria* sections

Our estimated crown ages for the four sections of *Myricaria* were about 8.83∼6.35 Ma (Figure 2), this is a remarkable molecular clock response to the rapid and major-range uplift of QTP at ca. 8 Ma. and the diversifications of four sections all occurred in the Himalayas (Figure 3): sections *Alpinae* and *Laxiflorae* in the eastern Himalayas (A), section *Parallelanthera* in western Himalayas (B), and section *Renantherae* in Himalayan union (AB). We have drawn the distinct morphological characters of four sections in Figure 2. This could be regarded as the response of four sections diversifications to the uplift in Himalayas.

The time of *Myricaria* origin and diversification related to Himalayan uplift in early Miocene, and the subsequent sectional diversifications at about 8.83∼6.35 Ma, correspond respectively to the two labels of the QTP uplift, Himalayan motion, and rapid and major-range uplift [21]–[29]. These diversification times of the genus and sections are very similar to those of the genus *Caragana* [38], which also has a 16 Ma time of generic origin and diversification ages of sections at about 8 Ma. However, here we provide the Himalayan places of origin and diversification for *Myricaria*, while those of *Caragana* remain unknown. The evolution of *Myricaria* is also temporally similar to that of the Asian spiny frogs *Paini* [42]. Uplift of the Himalayas, is hypothesized to be most likely due to a cut off of the genetic exchange at ca. 19 Ma, resulting in the splitting of the subgenera *Nanorana* and *Paa* of *Paini* began in the Miocene near 10 Ma [42].

### Migration along Asian mountains

For plant migration and dispersal, mountains generally act as a route or corridor [84], such as the Himalayan corridor mentioned above. Whereas the Himalayas for *Myricaria* are regarded as the center of origin, other distributions outside of the Himalayas and QTP can be understood as dispersals or migrations eastward, westward and northward along Asian mountains (see Figure 1). In fact, the results of vicariance and dispersal from biogeographical analysis (Figure 3) show that except for divergence of phylogenetically basal clades located in the eastern and western Himalayas (A and B), most remaining events, occurring in areas such as the Tianshan-Jungger-Turan (C), Altai-Sibiria (D), Mongolian Plateau (E), and Asia Minor-Caucasus-Europe (F) resulted from dispersal events. As evidenced from *Myricaria* (see Figure 1), dispersal and migration were possible from the Himalayas to Asia Minor-Caucasus-Europe as shown by *M. germanica*, and to the sand lands of the Mongolian Plateau by *M. bracteata*. These distributions along the Asian mountains are very similar to those of *Hippophae rhamnoides* (Elaeagnaceae) [37], a species with another interesting distribution in North Temperate Eurasia. *Hippophae rhamnoides* includes nine subspecies, and has been shown to have originated from the QTP, or more exactly, the eastern QTP-Hengduan Mountains, and then to have radiated and dispersed in different directions. Here, *Myricaria* originated in the Himalayas union, not in the eastern Himalayas only as *H. rhamnoides*. However, the northwestern Himalayas for *H. rhamnoides* and *Myricaria* played an important node role in connecting with Central Asia and Europe, and both dispersal route and direction is very similar.

## Supporting Information

Appendix S1 Two new sections within *Myricaria*.
(DOC)

## References

[1] Gaskin JF, Ghahremani-nejad F, Zhang DY, Londo JP (2004) A systematic overview of Frankeniaceae and Tamaricaceae from nuclear rDNA and plastid sequence data. Ann Mo Bot Gard 91: 401–409.

[2] Bremer B, Bremer K, Chase MW, Fay MF, Reveal JL, et al (2009) An update of the Angiosperm Phylogeny Group classification for the orders and families of flowering plants: APG III. Bot J Linn Soc 161: 105–121.

[3] Carlquist S (2010) Caryophyllales: a key group for understanding wood anatomy character states and their evolution. Bot J Linn Soc 164: 342–393.

[4] Zhang PY, Zhang YJ (1984) A study on the taxonomy of the genus *Myricaria* Desv. in China. Bul Bot Res 4: 67–80.

[5] Zhang PY, Zhang YJ (1990) Tamaricaceae. In: Flora Reipublicae Popularis Sinicae. Beijing: Science Press. pp. 142–177.

[6] Yang QE, Gaskin J (2007) Tamaricaceae. In: Wu ZY, Raven PH, editors. Flora of China, vol 13. Beijing/St. Louis: Science Press/Missouri Botanical Garden. pp. 58–66.

[7] Gorschkova S G (1949) Tamaricaceae. In: Komarov VL, editor. Flora of the USSR, vol 15. Moscow and Leningrad: Academiae Scientiarum URSS. pp. 290–327.

[8] Niedenzu F (1925) Tamaricaceae. In: Engler A, PrantlK, Die natürlichen Pflanzenfamilien.). Leipzig: Wilhelm Engelmann. pp. 276–281.

[9] Ovezinrlikov PN, Kinzikaeva GK (1977) *Myrtama* Ovez. et Kinz. gen. nov.: The new genus from the family Tamaricaceae Link. Dokl Akad Nauk USSR 20, 54–57.

[10] Zhang DY, Chen ZD, Sun HY, Yin L, Pan BR (2000) Systematic studies on some questions of Tamaricaceae based on ITS sequence. Acta Bot Bor-Occid Sin 20: 421–431.

[11] Zhang DY (2005) Discuss on some systematical problems of Tamaricaceae. Acta Bot Yunnan 27: 471–478.

[12] Hua L, Zhang DY, Pan BR (2004) Sequence analysis of the ITS region of nrDNA on Tamaricaceae from China and its systematic significance. Acta Bot Yunnan 26: 283–290.

[13] Wang Y, Liu YF, Liu SB, Huang HW (2009) Molecular phylogeny of *Myricaria* (Tamaricaceae): Implications for taxonomy and conservation in China. Bot Stud 50: 343–352.

[14] Liu YF, Wang Y, Huang HW (2009) Species-level phylogeographical history of *Myricaria* plants in the mountain ranges of western China and the origin of *M. laxiflora* in the Three Gorges mountain region. Mol Ecol 18: 2700–2712. 19457193 10.1111/j.1365-294X.2009.04214.x

[15] Liu YF, Wang Y, Huang HW (2006) High interpopulation genetic differentiation and unidirectional linear migration patterns in *Myricaria laxiflora* (Tamaricaceae), an endemic riparian plant in the Three Gorges Valley of the Yangtze River. Am J Bot 93: 206–215. 21646181 10.3732/ajb.93.2.206

[16] Wu ZY (1987) Origin and evolution of Xizang (Tibet) flora. In: Wu ZY, editor. Flora Xizangica. Beijing: Science Press. pp. 874–902.

[17] Willis KJ, McElwain JC (2002) The evolution of plants. Oxford: Oxford University Press.

[18] Guo Z, Sun B, Zhang Z, Peng S, Xiao G, et al (2008) A major reorganization of Asian climate by the early Miocene. Clim Past 4: 153–174.

[19] Qiang XK, An ZS, Song YG, Chang H, Sun YB, et al (2011) New eolian red clay sequence on the western Chinese Loess Plateau linked to onset of Asian desertification about 25 Ma ago. Sci China-Earth Sci 54: 136–144.

[20] Sun JM, Ye J, Wu WY, Ni XJ, Bi SD, et al (2010) Late Oligocene-Miocene mid-latitude aridification and wind patterns in the Asian interior. Geology 38: 515–518.

[21] Harris A, Xiang QY, Thomas DT (2009) Phylogeny, origin, and biogeographic history of *Aesculus* L.(Sapindales) an update from combined analysis of DNA sequences, morphology, and fossils. Taxon 58: 108–126.

[22] Wang CS, Zhao XX, Liu ZF, Lippert PC, Graham SA, et al (2008) Constraints on the early uplift history of the Tibetan Plateau. Proc Natl Acad Sci U S A 105: 4987–4992. 18362353 10.1073/pnas.0703595105 PMC2278176

[23] Najman Y, Pringle M, Johnson M, Robertson A, Wijbrans J (1997) Laser 40Ar/39Ar dating of single detrital muscovite grains from early foreland-basin sedimentary deposits in India: Implications for early Himalayan evolution. Geology 25: 535–538.

[24] Clark MK, House MA, Royden LH, Whipple KX, Burchfiel BC, et al (2005) Late Cenozoic uplift of southeastern Tibet. Geology 33: 525–528.

[25] Sun JM, Liu TS (2006) The age of the Taklimakan Desert. Science 312: 1621–1621. 16778048 10.1126/science.1124616

[26] Liu DL, Fang XM, Song CH, Dai S, Zhang T, et al (2010) Stratigraphic and paleomagnetic evidence of mid-Pleistocene rapid deformation and uplift of the NE Tibetan Plateau. Tectonophysics 486: 108–119.

[27] Miao YF, Herrmann M, Wu FL, Yan XL, Yang SL (2012) What controlled Mid-Late Miocene long-term aridification in Central Asia? - Global cooling or Tibetan Plateau uplift: A review. Earth Sci Rev 112: 155–172.

[28] Wang Y, Zheng J, Zhang W, Li S, Liu X, et al (2012) Cenozoic uplift of the Tibetan Plateau: Evidence from the tectonic-sedimentary evolution of the western Qaidam Basin. Geo Sci Front 3: 175–187.

[29] Molnar P (2005) Mio-pliocene growth of the Tibetan Plateau and evolution of East Asian climate. Palaeontol Electron 8: 1–23.

[30] Mao KS, Hao G, Liu JQ, Adams RP, Milne RI (2010) Diversification and biogeography of *Juniperus* (Cupressaceae): variable diversification rates and multiple intercontinental dispersals. New Phytol 188: 254–272. 20561210 10.1111/j.1469-8137.2010.03351.x

[31] Tu TY, Volis S, Dillon MO, Sun H, Wen J (2010) Dispersals of Hyoscyameae and Mandragoreae (Solanaceae) from the New World to Eurasia in the early Miocene and their biogeographic diversification within Eurasia. Mol Phylogen Evol 57: 1226–1237. 10.1016/j.ympev.2010.09.007 20858548

[32] Xu TT, Abbott RJ, Milne RI, Mao K, Du FK, et al (2010) Phylogeography and allopatric divergence of cypress species (*Cupressus* L.) in the Qinghai-Tibetan Plateau and adjacent regions. BMC Evol Biol 10(1): 194. 20569425 10.1186/1471-2148-10-194 PMC3020627

[33] Wu Z (1988) Hengduan mountain flora and her significance. J Jap Bot 63: 297–311.

[34] Liu J, Tian B (2007) Origin, evolution, and systematics of Himalaya endemic genera. Newslett Himalayan Bot 40: 20–27.

[35] Sun H (2002) Tethys retreat and Himalayas-Hengduanshan Mountains uplift and their significance on the origin and development of the Sino-Himalayas elements and alpine flora. Acta Bot Yunnan 24: 273–288.

[36] Kadereit JW, Licht W, Uhink CH (2008) Asian relationships of the flora of the European Alps. Plant Ecol Divers 1: 171–179.

[37] Jia DR, Abbott RJ, Liu TL, Mao KS, Bartish IV, et al (2012) Out of the Qinghai-Tibet Plateau: evidence for the origin and dispersal of Eurasian temperate plants from a phylogeographic study of *Hippophae rhamnoides* (Elaeagnaceae). New Phytol 194: 1123–1133. 22432741 10.1111/j.1469-8137.2012.04115.x

[38] Zhang ML, Fritsch PW (2010) Evolutionary response of *Caragana* (Fabaceae) to Qinghai-Tibetan Plateau uplift and Asian interior aridification. Plant Syst Evol 288: 191–199.

[39] Zhang ML, Fritsch PW, Cruz BC (2009) Phylogeny of *Caragana* (Fabaceae) based on DNA sequence data from *rbc*L, *trn*S-*trn*G, and ITS. Mol Phylogen Evol 50: 547–559. 10.1016/j.ympev.2008.12.001 19100848

[40] Rajbhandary S, Hughes M, Phutthai T, Thomas D, Shrestha KK (2011) Asian *Begonia*: out of Africa via the Himalayas? Gard Bull Singapore 63: 277–286.

[41] Zhou LL, Su YCF, Thomas DC, Saunders RMK (2012) Out-of-Africa' dispersal of tropical floras during the Miocene climatic optimum: evidence from *Uvaria* (Annonaceae). J Biogeogr 39: 322–335.

[42] Che J, Zhou WW, Hu JS, Yan F, Papenfuss TJ, et al (2010) Spiny frogs (*Paini*) illuminate the history of the Himalayan region and Southeast Asia. Proc Natl Acad Sci U S A 107: 13765–13770. 20643945 10.1073/pnas.1008415107 PMC2922240

[43] Doyle JJ, Doyle JL (1987) A rapid DNA isolation procedure for small quantities of fresh leaf tissue. Phytochem Bull 19: 11–15.

[44] Oxelman B, Liden M, Berglund D (1997) Chloroplast *rps*16 intron phylogeny of the tribe Sileneae (Caryophyllaceae). Plant Syst Evol 206: 393–410.

[45] Xu DH, Abe J, Sakai M, Kanazawa A, Shimamoto Y (2000) Sequence variation of non-coding regions of chloroplast DNA of soybean and related wild species and its implications for the evolution of different chloroplast haplotypes. Theor Appl Genet 101: 724–732.

[46] Johnson LA, Soltis DE (1995) Phylogenetic inference in Saxifragaceae sensu stricto and *Gilia* (Polemoniaceae) using *mat*K sequences. Ann Mo Bot Gard 82: 149–175.

[47] Thompson JD, Gibson TJ, Plewniak F, Jeanmougin F, Higgins DG (1997) The CLUSTAL_X windows interface: flexible strategies for multiple sequence alignment aided by quality analysis tools. Nucleic Acids Res 25: 4876–4882. 9396791 10.1093/nar/25.24.4876 PMC147148

[48] Farris JS, Källersjö M, Kluge AG, Bult C (1994) Testing significance of incongruence. Cladistics 10: 315–319.

[49] Drummond AJ, Ho SY, Phillips MJ, Rambaut A (2006) Relaxed phylogenetics and dating with confidence. PLoS Biol 4: e88. 16683862 10.1371/journal.pbio.0040088 PMC1395354

[50] Drummond AJ, Rambaut A (2007) BEAST: Bayesian evolutionary analysis by sampling trees. BMC Evol. Biol. 7: 214. 17996036 10.1186/1471-2148-7-214 PMC2247476

[51] Reveal JL (2011) Summary of recent systems of angiosperm classification. Kew Bull 66: 5–48.

[52] Anderson CL, Bremer K, Friis EM (2005) Dating phylogenetically basal eudicots using *rbc*L sequences and multiple fossil reference points. Am J Bot 92: 1737–1748. 21646091 10.3732/ajb.92.10.1737

[53] Magallón S, Castillo A (2009) Angiosperm diversification through time. Am J Bot 96: 349–365. 21628193 10.3732/ajb.0800060

[54] Bell CD, Soltis DE, Soltis PS (2010) The age and diversification of the angiosperms re-revisited. Am J Bot 97: 1296–1303. 21616882 10.3732/ajb.0900346

[55] Tiffney BH (1985) Perspectives on the origin of the floristic similarity between eastern Asia and eastern North America. J Arnold Arboret 66: 73–94.

[56] Wikström N, Savolainen V, Chase MW (2001) Evolution of the angiosperms: Calibrating the family tree. Proc Roy Soc B 268: 2211–2220. 10.1098/rspb.2001.1782 PMC1088868 11674868

[57] Lavin M, Herendeen PS, Wojciechowski MF (2005) Evolutionary rates analysis of Leguminosae implicates a rapid diversification of lineages during the Tertiary. Syst Biol 54: 575–594. 16085576 10.1080/10635150590947131

[58] Muller J (1981) Fossil pollen records of extant angiosperms. Bot Rev 47: 1–142.

[59] Eggens F (2006) Systematics in Sileneae (Caryophyllaceae) - Taxonomy and Phylogenetic patterns. Uppsala: Uppsala University.

[60] Fior S, Karis PO, Casazza G, Minuto L, Sala F (2006) Molecular phylogeny of the Caryophyllaceae (Caryophyllales) inferred from chloroplast *mat*K and nuclear rDNA ITS sequences. Am J Bot 93: 399–411. 21646200 10.3732/ajb.93.3.399

[61] Schuster TM, Setaro SD, Kron KA (2013) Age estimates for the buckwheat family Polygonaceae based on sequence data calibrated by fossils and with a focus on the Amphi-Pacific *Muehlenbeckia* . PloS One 8(4): e61261. 23585884 10.1371/journal.pone.0061261 PMC3621405

[62] Institute of Botany, NIoGaP, Academia Sinica (IB-NIGP) (1978) Neogene Floras. In Chinese Plant Fossils. Beijing: Academic Press.

[63] Ronquist F (1997) Dispersal - vicariance analysis: a new approach to the quantification of historical biogeography. Syst Biol 46: 195–203.

[64] Yu Y, Harris AJ, He X (2010) S-DIVA (statistical dispersalvicariance analysis): A tool for inferring biogeographic histories. Mol Phylogen Evol 56: 848–850. 10.1016/j.ympev.2010.04.011 20399277

[65] Ree RH, Smith SA (2008) Maximum likelihood inference of geographic range evolution by dispersal, local extinction, and cladogenesis. Syst Biol 57: 4–14. 18253896 10.1080/10635150701883881

[66] Ree RH, Moore BR, Webb CO, Donoghue MJ (2005) A likelihood framework for inferring the evolution of geographic range on phylogenetic trees. Evolution 59: 2299–2311. 16396171

[67] Ronquist F (1996) DIVA version 1.1. Computer program and manual. Available from: http://www.ebcuuse/systzoo/research/diva/divahtml.

[68] Nylander JAA, Olsson U, Alstrom P, Sanmartin I (2008) Accounting for phylogenetic uncertainty in biogeography: A Bayesian approach to dispersal - vicariance analysis of the thrushes (Aves: Turdus). Syst Biol 57: 257–268. 18425716 10.1080/10635150802044003

[69] Royle JF (1839) Illustration of the botany and other branches of the natural history of the Himalayan mountains, and of the Flora of Cashmerer. London: W.H. Aellen.

[70] Qaiser M, Ali S (1978) *Tamaricaria*, a new genus of Tamaricaceae. Blumea 24: 151–155.

[71] Baum B (1966) Monographic revision of the genus *Tamarix*. In: Final Research Report for the United States Department of Agriculture. pp. 141–142.

[72] Zhang ML (2003) Biogeography of *Astragalus* subgenus *Pogonophace* (Leguminosae). Acta Bot Yunnan 25: 25–32.

[73] Owen LA, Benn DI (2005) Equilibrium-line altitudes of the Last Glacial Maximum for the Himalaya and Tibet: an assessment and evaluation of results. Quatern Int 138: 55–78.

[74] Kitamura S (1955) Flowering plants and ferns. In: Kihara H, editor. Fauna and flora of Nepal Himalaya. Kyoto: Fauna and Flora Research Society. pp. 73–290.

[75] Tabata H (1988) On the Himalayan corridor. Acta Phytotax Geobot 39: 13–24.

[76] Tabata H (2004) On the Himalayan uplift and Himalayan corridors. Himalayan J Sci 2: 256–257.

[77] Xu LR, Zhang ML, Podlech D (2010) *Phyllolobium*. In: Wu ZY, Raven PH, editors. Flora of China. Beijing/St. Louis: Science Press/Missouri Botanical Garden Press. pp. 322–328.

[78] Zhang ML (1997) A reconstructing phylogeny in *Caragana* (Fabaceae). Acta Bot Yunnan 19: 331.

[79] Liu JQ, Gao TG, Chen ZD, Lu AM (2002) Molecular phylogeny and biogeography of the Qinghai-Tibet Plateau endemic *Nannoglottis* (Asteraceae). Mol Phylogen Evol 23: 307–325. 10.1016/s1055-7903(02)00039-8 12099790

[80] Liu JQ, Wang YJ, Wang AL, Hideaki O, Abbott RJ (2006) Radiation and diversification within the *Ligularia - Cremanthodium - Parasenecio* complex (Asteraceae) triggered by uplift of the Qinghai-Tibetan Plateau. Mol Phylogen Evol 38: 31–49. 10.1016/j.ympev.2005.09.010 16290033

[81] Wang YJ, Susanna A, Von Raab-Straube E, Milne R, Liu JQ (2009) Island-like radiation of *Saussurea* (Asteraceae: Cardueae) triggered by uplifts of the Qinghai-Tibetan Plateau. Biol J Linn Soc 97: 893–903.

[82] Wang YJ, Liu JQ, Miehe G (2007) Phylogenetic origins of the Himalayan endemic *Dolomiaea, Diplazoptilon* and *Xanthopappus* (Asteraceae: Cardueae) based on three DNA regions. Ann Bot 99: 311–322. 17218340 10.1093/aob/mcl259 PMC2802998

[83] Wang L, Abbott RJ, Zheng W, Chen P, Wang Y, et al (2009) History and evolution of alpine plants endemic to the Qinghai -Tibetan Plateau: *Aconitum gymnandrum* (Ranunculaceae). Mol Ecol 18: 709–721. 19175501 10.1111/j.1365-294X.2008.04055.x

[84] Wulff EV (1943) An introduction to historical plant geography. *An introduction to historical plant geography*.

