# Supplementary material for: Himalayan Origin and Evolution of Myricaria (Tamaricaeae) in the Neogene
Source: PLoS One. 2014 Jun 6;9(6):e97582. doi: 10.1371/journal.pone.0097582 (PMC4048171; doi:10.1371/journal.pone.0097582)
Supplement: Appendix S1 — Two new sections within Myricaria. (DOC) [file pone.0097582.s001.doc]

**Appendix S1** Two new sections within *Myricaria*.

**Section Alpinae** M.L. Zhang stat. nov.

Raceme lateral (or terminal), filaments ca. 2/3 united. Alpine, ca. 3000-5200 m altitude, endemic to Himalayas and Qinghai-Tibet Plateau, containing prostrate shrubs 5-14 cm tall (*M. prostrata*), recumbent shrubs ca. 1 m tall (*M. rosea*), and *M. wardii,* which even if an erect shrub, has smaller flowers ca. 5 mm long, and smaller bracts ca. 2-2.5 mm long. These distinct characters are likely adaptations to high altitude.

**Section Laxiforae** M.L. Zhang stat. nov.

Raceme terminal, lax (sparse), filaments ca. 1/3-1/2 united, bract margin narrow membranous, stamens length roughly equal to pistil. Endemic to the Three Gorges of the Yangtze River in Sichuan and Hubei Provinces of China. This section consists only of the endemic species *M. laxiflora*.
